# HLA Class I and Class II Associations with ESRD in Saudi Arabian Population

**DOI:** 10.1371/journal.pone.0111403

**Published:** 2014-11-07

**Authors:** Nuha Mahmoud Hamdi, Fadel Hassan Al-Hababi, Amr Ekhlas Eid

**Affiliations:** 1 Immunology Department, Riyadh Regional Laboratory, King Saud Medical Complex, Riyadh, Kingdom of Saudi Arabia; 2 Virology Department, Riyadh Regional Laboratory, King Saud Medical Complex, Riyadh, Kingdom of Saudi Arabia; 3 Nephrology Department, Riyadh Medical Complex, King Saud Medical Complex, Riyadh, Kingdom of Saudi Arabia; Temple University School of Medicine, United States of America

## Abstract

**Background:**

Chronic renal failure (CRF) leads in the majority of instances to end stage renal disease (ESRD) requiring renal replacement therapy. Our interest was to evaluate the possible associations of HLA class I and class II antigens with ESRD independent of other factors, in Saudi Arabia population.

**Methodology:**

A retrospective study to determine the HLA class I and class II polymorphisms and their association with ESRD, was performed on 350 patients with ESRD, and 105 healthy unrelated control. Patients and control groups were typed by SSOP lumenix techniques. The alleles positively associated to the ESRD were: HLA-B*15, B*18, B*49 - DRB1*03, negatively associated alleles were A*26, HLA-B*39, B*50. The haplotypes positively associated with ESRD were: HLA-A*01-DRB1*13 and HLA-A*30-DRBI*03. The negatively associated haplotypes were: HLA-A*02-B*39, A*02-B*50, A*24-B*35, A*24-B*58, A*24-DRB1*16, A*68-DRB1*04, A*02-DQB1*03, A*29-DQB1*02, A*29-DOB1*05 and B*27-DRB1*07 and the last one is the most significant protective haplotypes.

**Conclusion:**

The high Relative Risk (RR) observed and its statistical correlation reflect the strength of the described association between HLA antigens and ESRD.

## Introduction

Chronic Kidney Disease (CKD) is now one of the major health problems globally and early diagnosis is vital to prevent the development of end-stage renal failure (ESRD) which is defined as the need for renal replacement therapy such as dialysis or Kidney Transplant [1]. Various factors have been shown to be associated with ESRD such as age, gender, genetics, race, hypertension, and smoking etc. [2]. Although, the exact reasons for the growth of the ESRD program are unknown, it is postulated that changes in the demographics of the population, differences in disease burden among racial groups and under-recognition of earlier stages of CKD and of risk factors for CKD may partially explain this growth [3]. Genome-wide strategies have been used to pinpoint genes that contribute to the risks of development of these disorders [4].

In a recent systemic review based on 44 studies from Gulf Cooperation Council (GCC) showed that the incidence of ESRD has increased while the prevalence and mortality rate of ESRD in the GCC has not been reported sufficiently. The leading primary causes of ESRD recorded in the countries of GCC is diabetes which co-morbid conditions being hypertension and hepatitis C virus infection, the most common death was cardio vascular disease and sepsis [5]. The prevalence of CKD in the young Saudi population is around 5.7% [1]. According to the Saudi Center for Organ Transplantation (SCOT) annual report in 2012, the incidence of dialysis in the Kingdom of Saudi Arabia was 129 new cases per million population (PMP) with 3666 new patients in 2012 [6].

This compares to 360 PMP, 585 PMP and in the United States and Europe, respectively [7], [8]. While the prevalence of dialysis patients in the Kingdom of Saudi Arabia in 2012 was 499 new cases PMP with 14,171 dialysis patients [6]. This country showed higher prevalence that other developing countries which, it is vary from less than 100 PMP and 163 PMP in sub-Saharan Africa and India respectively, to about 330 PMP in Jordan and 360 PMP in Iran [9]–[11].

The rising prevalence is due largely to two main factors; the ageing of the population and the global epidemic of diabetes. An increasing prevalence of the most common causes of ESRD in the GCC: the prevalence of obesity that is associated with multi-chronic diseases have been reported, which exceeds that in the developed countries because of their rapid economic growth and associated changes in lifestyle [12], [13]. The International Diabetes Federation reports that five of the countries of the GCC ranked globally among the top ten countries in the world for diabetes prevalence [14].

The most common causes in Saudi Arabia is diabetic nephropathy (DN) prevalence as a cause of ESRD is 17.27%, glomerulonephritis 12.68%, hypertensive nephropathy prevalence is 7.75%, Secondary glomerulonephritis/Vasculitis haemolyticureamic syndrome (HUS) is 1.7%, uncertain aetiology, and other conditions, that are rarely reported as causes of ESRD, such as schistosomiasis, tuberculosis, sickle cell nephropathy and contrast nephropathy 19.59% [15]. Co-morbidities associated with ESRD diabetes prevalence is 47.85%, hypertension prevalence is 77.88%, cardiovascular disease prevalence is 14.51% and vascular disease as a co-morbidity condition as 11.7% [15].

Genes of human leukocyte antigens (HLA) (major histocompatability complex (MHC)) are located on short arm of chromosome six which are inherited in a co-dominant manner from parents to the offspring [16]. The HLA system is being paid more and more attention because it is very significant in polymorphous immunological reactions [17]. Histocompatibility testing has great importance in the selection of kidney recipient candidates and donors for transplantation, particularly HLA-DRB1, HLA-A, and HLA-B alleles, which code for molecules with a central role in the immune response and an essential role in the control of self recognition and consequently defense against microorganisms [18]. Previous studies have shown that certain polymorphisms of HLA class I and class II may influence the susceptibility to ESRD [19].

This study was conducted to determine protective and susceptible role of HLA class I and class II alleles and haplotypes in end stage renal disease patients.

## Materials and Methods

### Population Samples

This study investigated 350 unrelated Saudi patients were diagnosed with End Stage Renal disease (226 men/124 women, mean age ± SD, 43.2±16.67 years. For statistical comparison 105 healthy donors were recruited as control (58 men, 47 women, 30.3±15.43) from King Saud Medical City, King Fahad dialysis Center (table 1). HLA typing was performed in Department of immunology at Riyadh Regional Laboratory. Before launching into research, the research and clinical trials committee clearance from King Saud Medical City (KSMC).

**Table 1:** Characteristic of ESRD cases and controls for age and sex.

	ESRD	Controls
Gender (male/female)	226/124	58/47
Mean age ± SD	43.2±16.67	30.3±15.43

### DNA extraction and DNA typing of HLA loci

10 mL of blood was collected from the patients and normal donors after obtaining prior informed consent. DNA was isolated from blood samples by a fully automated system (MagNA Pure Compact, Roche) according to the manufacturer's instructions. The isolated DNA was stored at −80°C to be used for PCR. HLA-A, HLA-B HLA-DRB1 and DQRB1genotyping was performed by using LABType polymerase chain reaction reversed sequence-specific oligonucleotide (PCR-rSSO) (Luminex, One Lambda, Canoga Park, CA, USA) according to the manufacturer's instructions. Briefly, target DNA was amplified using specific primer sets that are biotinylated at 5 terminus for labeling the PCR product to allow its detection by R-Phycoerythrin-conjugated Streptavidine (SAPE). PCR product was denatured, neutralized, and rehybridized to oligonucleotide probes bound to fluorescently coded microbeads. Then biotinylated hybridized amplicons on microbeads were allowed to be labeled with SAPE. Luminex 100 flow analyzer detected the fluorescent intensities of Phycoerythrin (PE) on each microbeads. The retrieved output was analyzed by HLA Fusion software (ONE LAMBDA, INC, USA) for allele identification.

### Statistical analysis

HLA alleles and haplotype frequencies were estimated by direct counting in patients and in healthy controls to compare the differences between the allele frequencies in the control and ESRD groups, a 2×2 contingency table analysis was performed using the Fisher exact test. The strength of association between HLA alleles was estimated by Relative Risk (RR) and 95% confidence intervals (CI). P<0.05 was considered to be statistically significant and was considered sufficient to reject the null hypothesis. Values with relative risk higher than 1 were considered positive association with ESRD risk. Other alleles which express relative risk lowers than 1 were considered negative association with ESRD protection (A line on the software used for statistical analysis like SPSS, OpenEpi with Version no. 20).

## Results and Discussion

Since the mid-1980's, there has been marked increase in the incidence and prevalence of ESRD around the world [20]. Various pathologies may be associated to HLA alleles and/or haplotypes such as idiopathic membranous nephropathy, minimal change nephritic syndrome, immunoglobulin A (IgA) nephropathy, post-streptococcal glomerulonephritis and familiar focal glomerulonephritis [21], [22]. Family studies of the HLA class II system in acute post-streptococcal glomerulonephritis in the population of Zulia State confirm in the association between HLA alleles and the disease [23].

Idiopathic membranous glomerulonephritis is associated with HLA-DR2 in Japanese patients and with HLA-DR3 in Caucasians [24]. Similarly, compared to the Greek population, a higher frequency of HLA-DRB1*0301 has been reported in British patients with idiopathic membranous nephropathy [25]. Also anti-glomerular basement membrane nephritis in Japanese patients has a strong association with DRB1*1501 [26]. Although the presence of certain HLA alleles may be associated with increased susceptibility to renal disease, other HLA alleles may be protective, for example, DRB1*07 is negatively associated with membranous glomerulonephritis in Caucasians [27].

The present study showed that class I HLA-A*02 (ESRD 25.86% vs. con 26.67%) is the most frequent allele in ESRD and control groups of Saudi Arabian population. Other common alleles include A*01 (ESRD 10% vs. con 7.14%) (%), A*03 (ESRD 7.86% vs. con 6.19%), A*24 (ESRD 25.86% vs. con 26.67%), A*31 (ESRD 6.29% vs. con 7.62%), A*30 (ESRD 7% vs. con 4.76) and A*68 (ESRD 8.14% vs. con 10.48%) were the most frequent detected, with no significant difference between the ESRD patient and the control group. On the other hand HLA-A*26 (ESRD 5.14% vs. con 9.52%, p = 0.0318, RR = 0.54, CI = 0.3196 to 0.9123), showed a significant negative association with ESRD and emerged as a protective allele in ESRD (Table2).

**Table 2:** The frequency and associations of HLA A* alleles in ESRD patients and healthy controls.

Allele	ESRD N = 700	F	CON N = 210	F	P	RR	95% CI
HLA A*01	70	10.00	15	7.14	0.227	1.4	0.8192 to 2.3926
HLA A*02	181	25.86	56	26.67	0.8578	0.969643	0.7499 to 1.2538
HLA A*03	55	7.86	13	6.19	0.4588	1.269231	0.7075 to 2.2770
HLA A*06	1	0.14	0	0	1	0.903	0.0369 to 22.0856
HLA A*08	1	0.14	0	0	1	0.903	0.0369 to 22.0857
HLA A*11	13	1.86	7	3.33	0.2794	0.557143	0.2252 to 1.3784
HLA A*14	0	0	1	0.48	1	0.1003	0.0041 to 2.4540
HLA A*23	36	5.14	10	4.76	1	1.08	0.5453 to 2.1391
HLA A*24	58	8.29	17	8.10	1	1.023529	0.6097 to 1.7183
HLA A*26	36	5.14	20	9.52	0.0318	0.54	0.3196 to 0.9123
HLA A*29	10	1.43	5	2.38	0.3561	0.6	0.2074 to 1.7360
HLA A*30	49	7.00	10	4.76	0.3369	1.47	0.7579 to 2.8510
HLA A*31	44	6.29	16	7.62	0.526	0.825	0.4755 to 1.4314
HLA A*32	29	4.14	8	3.81	1	1.0875	0.5048 to 2.3426
HLA A*33	29	4.14	6	2.86	0.5393	1.45	0.6103 to 3.4452
HLA A*34	4	0.57	0	0.00	0.5789	2.709	0.1464 to 50.1147
HLA A*36	2	0.29	0	0	1	1.505	0.0725 to 31.2277
HLA A*62	1	0.14	0	0	1	0.903	0.0369 to 22.0856
HLA A*66	6	0.86	0	0.00	0.3547	3.913	0.2213 to 69.1773
HLA A*68	57	8.14	22	10.48	0.3275	0.777273	0.4872 to 1.2401
HLA A*69	2	0.29	0	0.00	1	1.505	0.0725 to 31.2277
HLA A*74	12	1.71	4	1.90	0.7714	0.9	0.2933 to 2.7613
HLA A*80	4	0.57	0	0	1	2.709	0.1464 to 50.1147
TOTAL	700	100.00	210	100.00			

N =  number of individuals, ESRD  =  End Stage Renal Disease, control  =  healthy donors, F =  alleles frequency, p =  Fisher's exact p value, RR =  Relative Risk.

The most common alleles in class I HLA B* alleles were B*07, (ESRD 8.29% vs. con 6.67%), B*08 (ESRD 8.86% vs. con 8.57), B*15 (ESRD 9.86% vs. con 3.81%, p = 0.0044), B*18 (ESRD 4.14% vs. con 0.95, p = 0.0277, B*35 (ESRD 6.43% vs. con 8.57%), B*39 (ESRD n = 11, 1.57%, vs. con 5.24%, p = 0.0076), B*49 (ESRD 4.29% vs. con 0.95%, p = 0.0181, RR = 4.5, CI = 1.0844 to 18.6739), B*50 (ESRD 11.57% vs. con 17.62% p = 0.026, and 51*(ESRD 15.86% vs. con 19.05%) in both ESRD patient and control group (Table 3). Positive significant difference for B*15 (RR = 2.5875, CI = 1.2650 to 5.2925), B*18 (RR = 4.35, CI = 1.0466 to 18.0795), B*49 as risk factor for ESRD and negative significant difference for B*39 (RR = 0.3, CI = 0.1319 to 0.6821), B*50 (RR = 0.656757, CI = 0.4596 to 0.9386) as a protective factor for ESRD were observed (Table 3).

**Table 3:** The frequency and associations of HLA B* alleles in ESRD patients and healthy controls.

Allele	ESRD N = 700	F	CON N = 210	F	P	RR	95% CI
HLA B*07	58	8.29	14	6.67	0.5598	1.242857	0.7079 to 2.1821
HLA B*08	62	8.86	18	8.57	1	1.033333	0.6257 to 1.7064
HLA B*13	14	2	5	2.38	0.7832	0.84	0.3061 to 2.3050
HLA B*14	14	2	3	1.43	0.775	1.4	0.4062 to 4.8253
HLA B*15	69	9.86	8	3.81	0.0044	2.5875	1.2650 to 5.2925
HLA B*18	29	4.14	2	0.95	0.0277	4.35	1.0466 to 18.0795
HLA B*27	8	1.14	6	2.86	0.1039	0.4	0.1404 to 1.1399
HLA B*31	1	0.14	0	0.00	1	0.903	0.0369 to 22.0856
HLA B*32	1	0.14	0	0.00	1	0.903	0.0369 to 22.0857
HLA B*35	45	6.43	18	8.57	0.2805	0.75	0.4439 to 1.2671
HLA B*37	5	0.71	1	0.48	1	1.5	0.1762 to 12.7686
HLA B*38	10	1.43	1	0.48	0.4725	3	0.3863 to 23.3007
HLA B*39	11	1.57	11	5.24	0.0076	0.3	0.1319 to 0.6821
HLA B*40	14	2	3	1.43	0.775	1.4	0.4062 to 4.8253
HLA B*41	18	2.57	9	4.29	0.2435	0.6	0.2736 to 1.3157
HLA B*42	11	1.57	1	0.48	0.3147	3.3	0.1540 to 0.8729
HLA B*44	19	2.71	6	2.86	1	0.95	0.3844 to 2.3479
HLA B*45	3	0.43	1	0.48	1	0.9	0.0941 to 8.6071
HLA B*47	3	0.43	0	0.00	1	2.107	0.1093 to 40.6298
HLA B*48	1	0.14	0	0.00	1	0.903	0.0369 to 22.0856
HLA B*49	30	4.29	2	0.95	0.0181	4.5	1.0844 to 18.6739
HLA B*50	81	11.57	37	17.62	0.026	0.656757	0.4596 to 0.9386
HLA B*51	111	15.86	40	19.05	0.2906	0.8325	0.6004 to 1.1544
HLA B*52	10	1.43	7	3.33	0.0839	0.428571	0.1652 to 1.1121
HLA B*53	25	3.57	5	2.38	0.5111	1.5	0.5814 to 3.8698
HLA B*55	3	0.43	1	0.48	1	0.9	0.0941 to 8.6071
HLA B*57	14	2	3	1.43	0.775	1.4	0.4062 to 4.8253
HLA B*58	26	3.71	6	2.86	0.6723	1.3	0.5424 to 3.1161
HLA B*73	2	0.29	1	0.48	1	0.6	0.0547 to 6.5845
HLA B*81	1	0.14			1	0.903	0.0369 to 22.0856
HLA B*92	1	0.14			1	0.903	0.0369 to 22.0856

N =  number of individuals, ESRD  =  End Stage Renal Disease, control  =  healthy donors, F =  alleles frequency, p =  Fisher's exact p value, RR =  Relative Risk.

In a study at the King Khalid University Hospital, which investigated 235 Saudi patients with ESRD and 60 normal, healthy individuals, the most common alleles in both groups were HLA-A2, A68, A24, A3, HLA-B51, B50, B7, B8 with no any significant difference between the two groups [20] [20]. Another report from the Azerbaijan republic, which investigated 26 patients with ESRD and 51 healthy controls, HLA-A2, A3, A24, and HLA-B35, B51 and B18 were the most frequently detected HLA antigens in all subjects. A significant association was found between susceptibility to ESRD and HLA-A11, A33 and HLA-B49 (P<0.05) [28].

In another study from Zulia and Venezuela on 188 patients with ESRD and 107 controls, HLA-B38, B51, B53 and B62 were positively associated with ESRD andHLA-A9, B12, B17, B40 and B48 were negatively associated with ESRD [23].

Diabetic nephropathy (DN) is leading cause of ESRD worldwide. It affects approximately 30% of patients with long-standing type 1 diabetes mellitus (T1DM) and type 2 diabetes mellitus (T2DM). Also familiar clustering of DN suggests genetic predisposition factor plays a role in the pathogenesis of this disease [29] [29]. Of the 12844 Saudi patients on dialysis, about 24% are in the active waiting list for transplantation with another 20% being evaluated for inclusion in the waiting list. The data showed that 31% of the dialysis patients are hypertensive, 14% are diabetic in addition to 30% suffer of both diabetes and hypertension [6].

For class II HLA-DRB1 our study showed that the most frequent alleles were DRB1*03 (ESRD n = 121, 17.29% vs. con n = 22, 10.48%), 04 (ESRD n = 99, 14.14% vs. con n = 40, 19.05), DRB1*07 (ESRD n = 110, 15.71 vs. con n = 43, 20.48), DRB1*11 (ESRD n = 71, 10.14% vs. con n = 15, 7.14%), DRB1*13 (ESRD n = 114, 16.29% vs. con n = 28, 13.33%) and DRB1*15 (ESRD n = 78, 11.14% vs. control, n = 27, 12.86%) in ESRD and control groups with no significant difference between the two group, except for DRB1*03 (p = 0.0173, RR = 1.65, CI = 1.0762 to 2.5296) as a risk factor for ESRD (Table 4). In HLA-DQB1 alleles showed common frequencies for DQB1*02 (ESRD n = 232, 33.14% vs. control n = 64, 30.77%), DQB1*03 (ESRD n = 167, 23.86% vs. control n = 61, 29.33%), DQB1*05 (ESRD n = 82, 11.71% vs. con n = 28, 13.46%) and DQB1*06 (ESRD n = 201, 28.71% vs. con n = 54, 25.96%) in ESRD and control groups with no significant difference (Table 5).

**Table 4:** The frequency and associations of HLA DRB1 alleles in ESRD patients and healthy controls.

Allele	ESRD N = 700	F	CON N = 210	F	P	RR	95% CI
DRB1*01	27	3.86	9	4.29	0.839	0.9	0.4301 to 1.8835
DRB1*02	1	0.14	0	0.00	1	0.903	0.0369 to 22.0856
DRB1*03	121	17.29	22	10.48	0.0173	1.65	1.0762 to 2.5296
DRB1*04	99	14.14	40	19.05	0.1003	0.7425	0.5321 to 1.0362
DRB1*05	1	0.14	0	0.00	1	0.903	0.0369 to 22.0856
DRB1*06	1	0.14	0	0.00	1	0.903	0.0369 to 22.0856
DRB1*07	110	15.71	43	20.48	0.1147	0.767442	0.5590 to 1.0537
DRB1*08	16	2.29	1	0.48	0.1416	4.8	0.6403 to 35.9829
DRB1*09	3	0.43	2	0.95	0.3266	0.45	0.0757 to 2.6753
DRB1*10	21	3	6	2.86	1	1.05	0.4294 to 2.5674
DRB1*11	71	10.14	15	7.14	0.2265	1.42	0.8315 to 2.4250
DRB1*12	5	0.71	1	0.48	1	1.5	0.1762 to 12.7686
DRB1*13	114	16.29	28	13.33	0.3304	1.221429	0.8323 to 1.7924
DRB1*14	7	1	3	1.43	0.7048	0.7	0.1826 to 2.6833
DRB1*15	78	11.14	27	12.86	0.538	0.866667	0.5754 to 1.3054
DRB1*16	25	3.57	13	6.19	0.1142	0.576923	0.3005 to 1.1076
TOTAL	**700**	**100**	**210**	**100**			

N =  number of individuals, ESRD  =  End Stage Renal Disease, control  =  healthy donors, F =  alleles frequency, p =  Fisher's exact p value, RR =  Relative Risk.

**Table 5:** The frequency and associations of HLA DQB1 alleles in ESRD patients and healthy controls.

Allele	ESRD N = 700	F	CON N = 210	F	P	RR	95% CI
DQB1*01	1	0.14	0	0	1	0.903	0.0369 to 22.0856
DQB1*02	232	33.14	64	30.77	0.5559	1.077143	0.8563 to 1.3549
DQB1*03	167	23.86	61	29.33	0.1216	0.813489	0.6342 to 1.0435
DQB1*04	13	1.86	1	0.48	0.21	3.862857	0.5107 to 29.4978
DQB1*05	82	11.71	28	13.46	0.545	0.870204	0.5832 to 1.2983
DQB1*06	201	28.71	54	25.96	0.4824	1.106032	0.8550 to 1.4308
DQB1*08	1	0.14	0	0	1	0.903	0.0369 to 22.0856
DQB1*20	1	0.14	0	0	1	0.903	0.0369 to 22.0856
DQB1*50	2	0.29	0	0	1	0.903	0.0369 to 22.0856
TOTAL	700	100.00	210	100			

N =  number of individuals, ESRD  =  End Stage Renal Disease, control  =  healthy donors, F =  alleles frequency, p =  Fisher's exact p value, RR =  Relative Risk.

This is in contrast to a study on Saudi population, which showed that in HLA-DRB1 *04, 07, 13, 03 (17), as the most frequently occurring class II alleles of both groups. While in HLA-DQB1 *02 and HLA-DQB1*06 were the most frequent alleles, with no significant difference between the groups except in DQB1*03 (8) (P = 0.04), which showed a positive association with ESRD [20].

The antigen frequencies were then compared to other studies on populations with close ethnicity Arab population. A recent study with Sudanese subjects showed that for the HLA-A loci, HLA-A2, 30, 3, 24, 1 and A68 were the most frequent antigens and for HLA-B loci HLA-B51, B41, B39, B57, B35, B50 and B52, were the most frequent antigens. HLA-DR13 and DR15 showed the highest antigen frequencies in the DR alleles. In HLA-DQ, DQ1 showed the highest gene frequency [30] [30]. In the Jordanian population A*0201 and B*0713, were the most frequent HLA class I alleles found and DRB1*0704, DRB1*0401 and DRB1*1501 were the most frequent [31] [31]. Another study on the HLA distribution in Egyptian patients with ESRD (n = 100) and normal healthy controls (n = 100), showed that the highest frequency for class I HLA-B35 (28%), B21 (18%) and class II HLA-DRB1*03 (29%) and DRB1*04 (29%). The number of individuals with HLA-B35 was significantly higher in the patient group (P = 0.025), whereas individuals with HLA-B5, B18 were significantly lower in the patient group (P = 0.044 and 0.048 respectively) [32].

This study was carried out without regard to primary renal disease leading to ESRD and focused on the final outcomes. This would help to avoid unexplained causes contributed by many known or unknown diseases. However, several studies provided the evidence that genetic polymorphism and epigenetic variations determine the individual susceptibility of patients to develop rapid progressive kidney disease [33]. A negative association between most A locus antigens and chronic glomerulonephritis and hypertensive nephrosclerosis were found In Turkish patients. And positive association were found between the HLA-B58 and HLA-DRB1*03 with amyloidosis and diabetic nephropathy, respectively [27]. Another Egyptian study among 457 individuals comprising 207 ESRD patients and 250 healthy controls showed that HLA-A2, -B8 and DRB1*3 and HLA-DRB1*11 were significantly correlated with diabetic nephropathy, in addition the B8-DR3 haplotype was shown to be susceptible to DM [34].

There is large variability in the frequency of HLA alleles present in the population ethnic groups. These differences in the results published by different authors are due to the variability in the frequency of HLA alleles present in the population ethnic groups. Also, may be due to the difference in the linkage disequilibrium between the HLA alleles and genes within or flanking the MHC region involved in immune response may play a role in ESRD progression. For instance the polymorphism in genes encoding the cytokines IL-6, IL-4 TNF-α and INF- γ have been shown that may be involved in the progression to ESRD [35]–[37].

Our study represents an expanded analysis for HLA haplotype among Saudi healthy population and its association in ESRD cases. Two loci heterogeneity haplotype, HLA A*02-B*51 (ESRD 4.58% vs. 6.03%) was the most common. A02- B50 (ESRD 3.35% vs. con 6.58%) and A02-B07 (ESRD 2.86% vs. con 2.47%) were less frequent, A02-DRB113 (ESRD 4.33% vs. con 2.48%) and A02-DRB1 03 (ESRD 4.08% vs. con 6.61%), A02-DQB102 (ESRD 8.49% vs. con 9.64%) and A02-DQB1 06 (ESRD 7.35% vs. con 7.44%) (Tables: 6, 7 and 8). Furthermore, B08-DRB1 03 (ESRD 3.67% vs. con 2.53%) and B51-DRB1 13 (ESRD 3.29% vs. con 3.93%) are the common haplotypes (Tables 9 and 10). However, no significant difference between the ESRD patients and control haplotypes were observed.

**Table 6:** Main high-frequency HLA A–B haplotype and their frequencies in ESRD patients and healthy controls (haplotype frequency ≥1.5%).

Haplotype	ESRD	ESRD %	CONT	CONT %	P value	RR	95% CI
A1-B51	24	1.96	6	1.64	0.8285	1.1938	0.4917 to 2.8981
A2-B07	35	2.86	9	2.47	0.8559	1.1606	0.5632 to 2.3919
A2-B15	20	1.64	2	0.55	0.1978	2.9845	0.7009 to 12.7087
A2-B35	18	1.47	8	2.19	0.074	0.6715	0.2944 to 1.5317
A2-B39	7	0.57	7	1.92	0.0242	0.2984	0.1054 to 0.8453
A2-B50	41	3.35	24	6.58	0.0098	0.5098	0.3123 to 0.8322
A2-B51	56	4.58	22	6.03	0.2701	0.7597	0.4705 to 1.2266
A3-B50	19	1.55	3	0.82	0.4435	1.8902	0.5625 to 6.3516
A24-B08	21	1.72	4	1.1	0.482	1.5668	0.5413 to 4.5354
A24-B35	10	0.82	8	2.19	0.0441	0.3731	0.1483 to 0.9383
A24-B08	19	1.55	9	2.47	0.258	0.6301	0.2875 to 1.3806
A24-B58	1	0.08	4	1.1	0.0113	0.0746	0.0084 to 0.6655
A31-B50	11	0.9	7	1.92	0.1528	0.469	0.1831 to 1.2011
A31-B51	20	1.64	12	3.29	0.0566	0.4974	0.2455 to 1.0078
A68-B51	20	1.64	6	1.64	1	0.9948	0.4025 to 2.4587

**Table 7:** Main high-frequency HLA A – DRB1 haplotype and their frequencies in ESRD patients and healthy controls (haplotype frequency ≥1.5%).

HAPLOTYPE	ESRD	ESRD %	CONT	CONsT %	P value	RR	95% CI
A*01-DRB1*03	19	1.55	3	0.83	0.6261	1.8783	0.5590 to 6.3115
A*01-DRB1*07	21	1.72	6	1.65	0.4432	1.038	0.4221 to 2.5523
A*01-DRB1*13	20	1.63	0	0	0.0123	12.1829	0.7386 to 200.9482
A*01-DRB1*15	15	1.23	6	1.65	0.6002	0.7414	0.2898 to 1.8971
A*02 -DRB1*03	45	3.68	8	2.2	0.1872	1.6682	0.7936 to 3.5065
A*02 -DRB1*04	40	3.27	18	4.96	0.1507	0.659	0.3826 to 1.1352
A*02 -DRB1*07	50	4.08	24	6.61	0.064	0.6179	0.3852 to 0.9910
A*02 -DRB1*11	22	1.8	6	1.65	1	1.0874	0.4443 to 2.6615
A*02 -DRB1*13	53	4.33	9	2.48	0.1238	1.7465	0.8700 to 3.5059
A*02 -DRB1*15	36	2.94	17	4.68	0.1324	0.628	0.3570 to 1.1047
A*02 -DRB1*16	16	1.31	7	1.93	0.4512	0.628	0.3570 to 1.1047
A*03 -DRB1*03	19	1.55	3	0.83	0.6261	1.8783	0.5590 to 6.3115
A*03 -DRB1*04	12	0.98	6	1.65	1	0.5965	0.2255 to 1.5783
A*24 -DRB1*03	30	2.45	4	1.1	0.1486	2.2243	0.7888 to 6.2721
A*24 -DRB1*04	20	1.63	7	1.93	0.6495	0.8473	0.3612 to 1.9879
A*24 -DRB1*16	3	0.25	6	1.65	0.0061	0.1483	0.0373 to 0.5900
A*26 -DRB1*03	19	1.55	11	3.03	0.0793	0.5123	0.2461 to 1.0665
A*26 -DRB1*04	15	1.23	7	1.93	0.311	0.6355	0.2611 to 1.5467
A*30 -DRB1*03	20	1.63	0	0	0.0123	12.1829	0.7386 to 200.9482
A*31-DRB1*13	28	2.29	10	2.75	0.5631	0.8304	0.4072 to 1.6932
A*68-DRB1*04	19	1.55	12	3.31	0.049	0.4696	0.2301 to 0.9581
A*68-DRB1*07	18	1.47	7	1.93	0.4821	0.7626	0.3210 to 1.8115
A*68-DRB1*15	10	0.82	6	1.65	0.2255	0.4943	0.1809 to 1.3508

**Table 8:** Main high-frequency HLA A – DQB1 haplotype and their frequenciesin ESRD patients and healthy controls (haplotype frequency ≥1.5%).

HAPLOTYPE	ESRD	ESRD %	CONT	CONT %	P value	RR	95% CI
A*01-DQB1*02	36	2.94	6	1.65	0.2620	1.6438	0.6981 to 3.8707
A*01-DQB1*03	31	2.53	10	2.75	0.8505	0.8493	0.4204 to 1.7159
A*01-DQB1*06	38	3.1	6	1.65	0.2005	1.8767	0.7997 to 4.4042
A*02-DQB1*02	104	8.49	35	9.64	0.5257	0.8805	0.6115 to 1.2680
A*02-DQB1*03	56	4.57	27	7.44	0.0429	0.6146	0.3942 to 0.9582
A*02-DQB1*05	35	2.86	10	2.75	1.0000	1.0371	0.5187 to 2.0739
A*02-DQB1*06	90	7.35	27	7.44	1.0000	0.9878	0.6530 to 1.4941
A*03-DQB1*02	24	1.96	5	1.38	0.6552	1.4224	0.5466 to 3.7014
A*03-DQB1*03	23	1.88	8	2.2	0.6684	0.8519	0.3844 to 1.8883
A*03-DQB1*06	21	1.71	6	1.65	1.0000	1.0371	0.4218 to 2.5502
A*11-DQB1*02	24	1.96	5	1.38	0.6552	1.4224	0.5466 to 3.7014
A*11-DQB1*03	20	1.63	4	1.1	0.6262	1.4816	0.5097 to 4.3071
A*24-DQB1*02	43	3.51	9	2.48	0.4028	1.4158	0.6969 to 2.8764
A*24-DQB1*03	27	2.2	10	2.75	0.5531	0.8001	0.3910 to 1.6372
A*24-DQB1*05	13	1.06	8	2.2	0.1140	0.4815	0.2011 to 1.1527
A*24-DQB1*06	18	1.47	7	1.93	0.4818	0.762	0.3208 to 1.8100
A*26-DQB1*02	25	2.04	14	3.86	0.0549	0.5292	0.2780 to 1.0072
A*26-DQB1*03	19	1.55	9	2.48	0.2412	0.6256	0.2855 to 1.3707
A*26-DQB1*06	16	1.31	7	1.93	0.4511	0.6773	0.2808 to 1.6337
A*29-DQB1*02	4	0.33	5	1.38	0.0337	0.2371	0.0640 to 0.8782
A*29-DQB1*05	1	0.08	5	1.38	0.0030	0.0593	0.0069 to 0.5057
A*30-DQB1*02	29	2.37	8	2.2	1.0000	1.0742	0.4954 to 2.3291
A*30-DQB1*03	21	1.71	3	0.83	0.3267	2.0743	0.6222 to 6.9149
A*30-DQB1*05	13	1.06	2	0.55	0.5422	1.9261	0.4367 to 8.4962
A*30-DQB1*06	29	2.37	3	0.83	0.0864	2.8645	0.8776 to 9.3493
A*31-DQB1*02	23	1.88	8	2.2	0.6684	0.8519	0.3844 to 1.8883
A*31-DQB1*03	14	1.14	8	2.2	0.1308	0.5186	0.2193 to 1.2264
A*31-DQB1*06	44	3.59	14	3.86	0.8734	0.9313	0.5163 to 1.6799
A*32-DQB1*02	23	1.88	4	1.1	0.4867	1.7039	0.5931 to 4.8951
A*32-DQB1*03	15	1.22	7	1.93	0.3107	0.635	0.2609 to 1.5455
A*33-DQB1*02	21	1.71	2	0.55	0.1334	3.1114	0.7330 to 13.2073
A*68-DQB1*02	33	2.69	8	2.2	0.7087	1.2223	0.5697 to 2.6228
A*68-DQB1*03	30	2.45	14	3.86	0.1490	0.635	0.3404 to 1.1845
A*68-DQB1*06	25	2.04	10	2.75	0.4174	0.7408	0.3592 to 1.5280

**Table 9:** Main high-frequency HLA B– DRB1 haplotype and their frequencies in ESRD patients and healthy controls (haplotype frequency ≥1.5%).

HAPLOTYPE	ESRD	ESRD %	CONT	CONT%	P value	RR	95% CI
B*07-DRB1*13	23	1.76	3	0.84	0.333	2.0882	0.6306 to 6.9154
B*07-DRB1*15	25	1.91	8	2.25	0.669	0.8512	0.3873 to 1.8708
B*08-DRB1*03	48	3.67	9	2.53	0.3289	1.4527	0.7198 to 2.9319
B*08-DRB1*04	13	0.99	6	1.69	0.2671	0.5902	0.2259 to 1.5417
B*14-DRB1*03	23	1.76	3	0.84	0.333	2.0882	0.6306 to 6.9154
B*14-DRB1*13	34	2.6	7	1.97	0.5684	1.323	0.5915 to 2.9592
B*27-DRB1*07	0	0	5	1.4	0.0004	0.0248	0.0014 to 0.4477
B*35-DRB1*04	18	1.38	9	2.53	0.1528	0.5448	0.2469 to 1.2022
B*35-DRB1*16	3	0.23	6	1.69	0.0043	0.1362	0.0342 to 0.5419
B*50-DRB1*04	21	1.61	13	3.65	0.0316	0.44	0.2225 to 0.8700
B*50-DRB1*07	66	5.05	28	7.87	0.0513	0.642	0.4192 to 0.9833
B*50-DRB1*13	24	1.84	2	0.56	0.0948	3.2686	0.7762 to 13.7646
B*51-DRB1*03	20	1.53	5	1.4	1	1.0895	0.4118 to 2.8826
B*51-DRB1*04	36	2.75	12	3.37	0.5991	0.8171	0.4297 to 1.5539
B*51-DRB1*07	26	1.99	6	1.69	0.8302	1.1803	0.4896 to 2.8454
B*51-DRB1*13	43	3.29	14	3.93	0.5153	0.8366	0.4630 to 1.5116
B*51-DRB1*15	31	2.37	11	3.09	0.447	0.7676	0.3897 to 1.5119
B*52-DRB1*15	7	0.54	7	1.97	0.0165	0.2724	0.0962 to 0.7715
B*52-DRB1*03	16	1.22	3	0.84	0.7793	1.4527	0.4256 to 4.9579

**Table 10:** Main high-frequency HLA B– DQB1 haplotype and their frequencies in ESRD patients and healthy controls (haplotype frequency ≥1%).

HAPLOTYPE	ESRD	ESRD %	CONT	CONT %	P value	RR	95% CI
B*07-DQB1*02	22	2.83	6	1.69	1	0.9987	0.4081 to 2.4444
B*07-DQB1*03	29	1.3	8	2.25	1	0.9874	0.4554 to 2.1409
B*07-DQB1*06	37	0.31	10	2.82	1	1.0078	0.5062 to 2.0066
B*08-DQB1*02	48	4.29	13	3.67	1	1.0057	0.5511 to 1.8353
B*08-DQB1*03	22	1	6	1.69	1	0.9987	0.4081 to 2.4444
B*08-DQB1*06	33	2.14	9	2.54	1	0.9987	0.4824 to 2.0676
B*15-DQB1*02	11	2.22	3	0.85	1	0.9987	0.2801 to 3.5607
B*15-DQB1*06	33	3.6	9	2.54	1	0.9987	0.4824 to 2.0676
B*35-DQB1*02	15	1.91	4	1.13	1	1.0214	0.3411 to 3.0585
B*35-DQB1*03	55	2.07	15	4.24	1	0.9987	0.5712 to 1.7463
B*35-DQB1*05	30	0.31	8	2.26	1	1.0214	0.4724 to 2.2084
B*35-DQB1*06	18	1.84	5	1.41	1	0.9806	0.3666 to 2.6228
B*39-DQB1*02	22	0.31	6	1.69	1	0.9987	0.4081 to 2.4444
B*41-DQB1*03	22	1.61	6	1.69	1	0.9987	0.4081 to 2.4444
B*50-DQB1*02	122	5.44	33	9.32	1	1.007	0.6981 to 1.4524
B*50-DQB1*03	44	2.07	12	3.39	1	0.9987	0.5333 to 1.8705
B*50-DQB1*06	22	2.76	6	1.69	1	0.9987	0.4081 to 2.4444
B*51-DQB1*02	48	3.83	13	3.67	1	0.9987	0.5333 to 1.8705
B*51-DQB1*03	74	3.98	20	5.65	1	1.0927	0.6757 to 1.7672
B*51-DQB1*05	18	1.99	5	1.41	1	0.9806	0.3666 to 2.6228
B*51-DQB1*06	96	5.67	26	7.34	1	1.0057	0.6627 to 1.5263
B*52-DQB1*06	26	0.69	7	1.98	1	1.0117	0.4428 to 2.3116
B*58-DQB1*02	15	2.14	4	1.13	1	1.0214	0.3411 to 3.0585

However, the haplotypes that showed statistically significant values with negative association with ESRD protection were A2-B50 (p = 0.0098, RR = 0.5098, CI = 0.3123 to 0.8322), A24-B35 (p = 0.0441, RR = 0.3731, CI = 0.1483 to 0.9383), A*68-DRB1*04 (p = 0.049, RR = 0.4696, CI = 0.2301 to 0.9581), A*02-DQB1*03 (p = 0.0429, RR = 0.6146, CI = 0.3942 to 0.9582). The A01-DRB1 13 (p = 0.0123, RR = 12.1829, CI = 0.7386 to 200.9482) and A30 -DRB1 03 (p = 0.0123, RR = 12.1829, CI = 0.7386 to 200.9482) showed statistically significant values with positive association with ESRD risk.

The haplotypes investigation of ESRD from Zulia, Venezuela population showed positive association with ESRD in: A2-B51, A2-B53, A23-B38 and A68-B38. The A42-B12, A9-B35 and A28-B40 haplotypes were negatively associated with ESRD, expressing relative risk lower than 1 [16]. On the correlations between HLA haplotype and various forms of glomerulopathy, HLA-A2, -B8 and DRB1*3 and HLA-DRB1*11 significantly correlated with diabetic nephropathy, respectively. B8-DR3 haplotype is susceptible to DM [34].

A study investigating the incidence of kaposi's sarcoma after renal transplantation reported a remarkably high frequency of HLA-A2 (83.3%) among patients compared to normal individuals (43.6%) [38]. In addition, a Canadian study found a higher percentage (69%) of the HLA-A2 allele in young patients with diabetic ESRD compared to normal, healthy individuals (36%), with the majority of patients showing either the HLA-A2-DR4 or HLA-DR8 haplotypes.

In developing countries like Saudi Arabia and other Gulf Cooperation Council (GCC) countries, which share a similar cultural background, ethnicity, socio-demographic distributions and socioeconomic development, many reports, has been shown evidence of increasing prevalence of the most common causes of ESRD [5]. Also there is increasing in the prevalence of obesity in these countries and its associated multichronic diseases, exceeds that in the developed countries because of their rapid economic growth and associated changes in lifestyle [12], [13]. Since certain haplotypes particularly two loci and three loci are common to some of the racial groups, it would be possible to find small disparity between population within these countries in the HLA frequencies and haplotype. Hence, there is a need to compare haplotype frequencies among the populations from different regions of Saudi Arabia and GCC. This would be important for creating a national registry and organ-sharing network for GCC people.

In conclusion, this study throws light on the frequency and distribution of HLA class I and class II alleles and haplotypes that confer susceptibility and protection in the Saudi Arabian patients with end stage renal disease (ESRD). However a larger study encompassing more number of patients and controls would be needed to assess the clinical relevance of the results obtained. In the future, molecular diagnostics on HLA polymorphisms may serve to identify patients with survival advantages and could be used by physicians to plan their treatment in the GCC.

## Recommendation

The association of HLA alleles and/or haplotype could be used as a risk classification marker and thereby progression to much more severe disease could be protected or diagnosed earlier. This would require further investigation with larger number of patients and healthy individuals and with every disease may cause ESRD. A great need to compare haplotype frequencies among the populations from different regions of Saudi Arabia and GCC populations to develop a national registry and organ-sharing network for GCC people.
