# Correction: HLA Class I and Class II Associations with ESRD in Saudi Arabian Population

**DOI:** 10.1371/journal.pone.0190127

**Published:** 2017-12-18

**Authors:** Nuha Mahmoud Hamdi, Fadel Hassan Al-Hababi, Amr Ekhlas Eid

There is an affiliation missing for the first author. Nuha Mahmoud Hamdi should also be affiliated with: Immunology and Clinical Pathology Department, Faculty of Medicine (for girls), Al-Azhar University, Cairo, Egypt.
